# Trans-sialidase-based vaccine candidate protects against *Trypanosoma cruzi* infection, not only inducing an effector immune response but also affecting cells with regulatory/suppressor phenotype

**DOI:** 10.18632/oncotarget.18217

**Published:** 2017-05-25

**Authors:** Estefanía Prochetto, Carolina Roldán, Iván A. Bontempi, Daiana Bertona, Luz Peverengo, Miguel H. Vicco, Luz M. Rodeles, Ana R. Pérez, Iván S. Marcipar, Gabriel Cabrera

**Affiliations:** ^1^ Facultad de Bioquímica y Ciencias Biológicas, Universidad Nacional del Litoral, Santa Fe, Argentina; ^2^ Facultad de Ciencias Médicas, Universidad Nacional del Litoral, Santa Fe, Argentina; ^3^ IDICER-CONICET and Instituto de Inmunología, Facultad de Ciencias Médicas, Universidad Nacional de Rosario, Santa Fe, Argentina

**Keywords:** Trypanosoma cruzi, trans-sialidase, vaccine, foxp3 regulatory T cells, myeloid-derived suppressor cells, Immunology and Microbiology Section, Immune response, Immunity

## Abstract

Prophylactic and/or therapeutic vaccines have an important potential to control *Trypanosoma cruzi* (*T. cruzi*)infection. The involvement of regulatory/suppressor immune cells after an immunization treatment and *T. cruzi* infection has never been addressed. Here we show that a new trans-sialidase-based immunogen (TSf) was able to confer protection, correlating not only with beneficial changes in effector immune parameters, but also influencing populations of cells related to immune control.

Regarding the effector response, mice immunized with TSf showed a TS-specific antibody response, significant delayed-type hypersensitivity (DTH) reactivity and increased production of IFN-γ by CD8+ splenocytes. After a challenge with *T. cruzi*, TSf-immunized mice showed 90% survival and low parasitemia as compared with 40% survival and high parasitemia in PBS-immunized mice.

In relation to the regulatory/suppressor arm of the immune system, after *T. cruzi* infection TSf-immunized mice showed an increase in spleen CD4+ Foxp3+ regulatory T cells (Treg) as compared to PBS-inoculated and infected mice. Moreover, although *T. cruzi* infection elicited a notable increase in myeloid derived suppressor cells (MDSC) in the spleen of PBS-inoculated mice, TSf-immunized mice showed a significantly lower increase of MDSC.

Results presented herein highlight the need of studying the immune response as a whole when a vaccine candidate is rationally tested.

## INTRODUCTION

Chagas disease is caused by the protozoan parasite *Trypanosoma cruzi* (*T. cruzi*). About 7 million people are estimated to be infected in Latin America, and international migration is increasing the number of *T. cruzi* infected individuals in non-endemic countries [1], [2]. Although Chagas disease can be treated with benznidazole or nifurtimox during the acute phase of the disease, the efficiency of both drugs is still unclear in chronic patients, who may suffer some adverse reactions, occurring in up to 40% of treated patients [3], [4]. Moreover, a recent multicenter trial has shown that chemotherapeutic treatment dampens parasite load but does not decrease morbility nor mortality associated with the Chagas disease cardiomyopathy [5]. Thus, prophylactic and therapeutic vaccines would be suitable alternatives for preventing and/or treating Chagas disease.

Several studies have focused on characterization of parasite antigens which may be used as vaccine candidates. Some recombinant or purified antigens have shown promising results in mouse models, such as paraflagellar rod proteins, trypomastigote excretory-secretory antigens, glycoprotein 82, trypomastigote surface antigen 1, Tc52, cruzipain and trans-sialidase (TS) [6], [7], [8], [9], [10], [11], [12].

In particular, TS is a multifunctional protein that has a pivotal role in *T. cruzi* infection. It has been shown that TS scavenges sialic acid from the host allowing the parasite to avoid lysis by serum factors and to interact and invade mammalian host cells [13], [14]. Studies from our group and others have shown that vaccines candidates based on TS are able to generate protection against *T. cruzi* infection [11], [15], [16], [17], [18].

It is well accepted that components of a T helper 1 (Th1) type immune response are required to control parasite infection [19], [20], [21], [22]. Taking into account that it has been reported that immunization with TS alone, without any adjuvant, inhibits the development of a Th1 type response, the use of a proper adjuvant is necessary to redirect the response to a Th1 profile [11], [23]. For this purpose, we have previously employed ISCOMATRIX (IMX) adjuvant [11] and now we have developed a new adjuvant composed of lipidic cages (ISPA, manuscript in preparation) that shows similar activity to IMX concerning the elicitation of a response that includes several components of the Th1 profile.

On the other hand, cumulative evidence strongly supports that vaccines may influence not only the effector arm of the immune system, but also the regulatory/suppressor counterpart [24], [25], [26], [27]. Despite this evidence, few vaccines studies have addressed this issue by evaluating alterations in cells with immunomodulatory capacities, such as Foxp3+ regulatory T cells (Treg) or myeloid-derived suppressor cells (MDSC). Treg cells, which constitute around 10% of peripheral CD4+ T cells, have a potent anti-inflammatory effect that is essential for maintaining immune homeostasis [28]. On the other hand, MDSC is a heterogeneous population comprising monocytes, granulocytes and dendritic cells at different stages of differentiation, in all cases expressing markers like CD11b and GR-1 (Ly6C/Ly6G)+ [29]. In the particular case of Chagas disease, Tregs and MDSC cells may have strong relevance because it has been shown that immunomodulation plays a critical role during both the acute and the chronic phase of the disease. For instance, it has been reported that *T. cruzi* infection elicits an important increase of spleen MDSC cells during the acute phase [30], [31], while the role of Treg cells remains to be completely elucidated during the acute and the chronic phase of the disease [32], [33], [34], [35], [36], [37].

We have already shown that immunization with a recombinant full-length TS antigen protected against *T. cruzi* infection [11]. However, the availability of a TS of reduced size, and similar protective capacity, would represent a valuable tool for vaccine development taking in mind that heterologous expression of antigens is improved when DNA sequence size is lower than 1000 base pairs [38]. Such immunogen could be useful not only for immunization with a subunit formulation, but also for cloning into recombinant bacteria designed to be used as delivery system.

Taking together, the aim of this work was to obtain a reduced TS fraction with protecting capacity against *T. cruzi*, and assess whether the new immunogen influenced the effector response as well as the regulatory/suppressor arm of the immune system.

## RESULTS

### Development of a TSf fraction protein

Several issues were taken in mind in order to obtain a TS fraction: a) the most beneficial size for cloning into recombinant bacteria, b) inactivation of the enzyme by excluding a complete domain, c) exclusion of the SAPA domain. According to the tasks considered and methodological conditions, a TS fragment (TSf) that included the region from the aminoacid 338 to 627 was selected to use.

It has been previously shown that the TS sequence indexed in the GenBank AJ276679 codifies for a TS protein that has protective capacity against *T. cruzi* infection [15]. Thus, the primers for the amplification of the TSf fragment were designed considering the sequence AJ276679.

**Figure 1:**
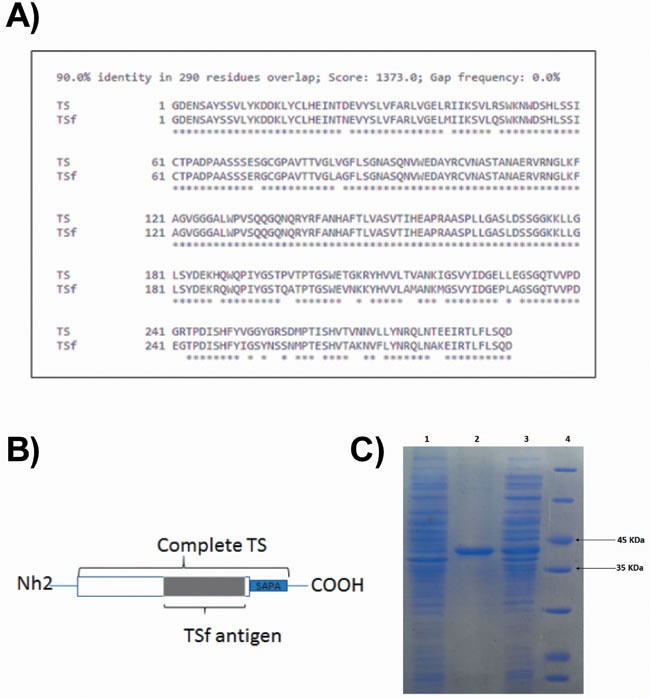
Development of the TSf fragment **A.** The TSf fragment obtained showed 90% aminoacidic identity as aligned with the TS indexed in the GenBank AJ276679 [15]. **B.** A representative picture of the TSf fragment as compared to the full-length TS protein. **C.** Assessment of TSf protein purification on SDS polyacrylamide gel electrophoresis: lane 1 non-induced colony, lane 2: TSf protein that was purified in Ni-NTA resin-based affinity chromatography and dialyzed against urea 0,5M; lane 3: TSf protein expressed but not purified, lane 4: low molecular weight marker.

A desirable fragment of 884 bp was obtained by PCR. The aminoacidic translation showed 90% identity against the TS protein corresponding to the DNA sequence used to design the primers (Figure 1A). As expected by the construction, the TS fragment obtained (TSf) aligned with the middle portion of the TS protein, as shown in Figure 1B.

After protein production and purification, the presence and purity of TSf were determined on SDS-PAGE (Figure 1C).

### TSf-ISPA immunized mice showed immunological parameters that included components of a Th1 response

We have recently developed an adjuvant composed of lipidic cages (ISPA) that induces a balanced immune response with high antibodies titers against several and diverse antigens (manuscript in preparation).

**Figure 2:**
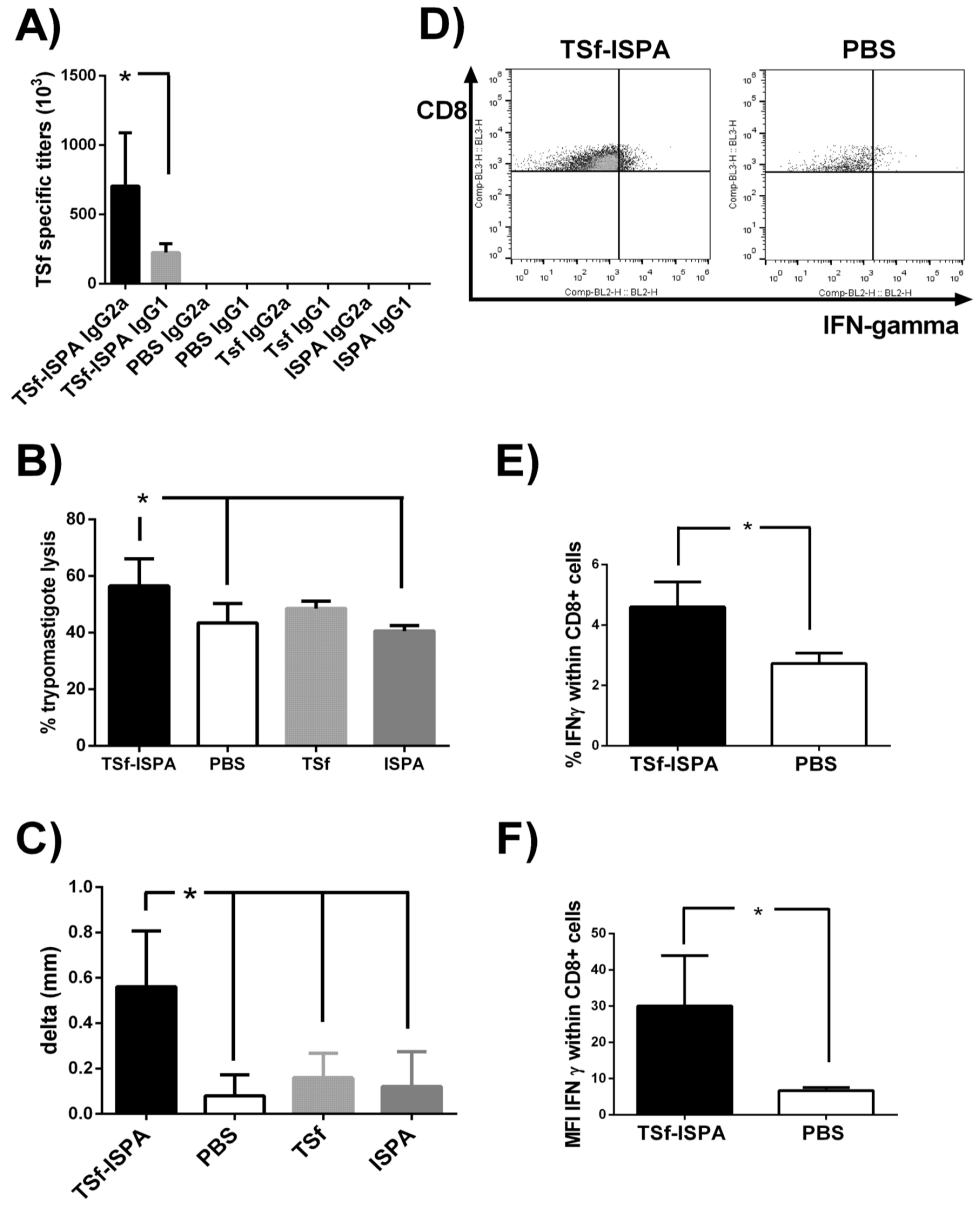
Immunological parameters of the immune response elicited by TSf-ISPA immunization **A.** BALB/c mice were immunized with TSf-ISPA, PBS, ISPA-alone or TSf-alone and plasma samples were analyzed for TSf-specific IgG2a and IgG1 antibodies titers by ELISA. Antibodies titers were negligible in plasma from PBS, TSf-alone or ISPA-alone immunized mice. **B.** Trypomastigote lysis assay was performed using serum from TSf-ISPA, ISPA-alone, TSf-alone and PBS-inoculated mice. **C.** DTH response in immunized mice: Footpad thickness was measured before and 48 h after inoculation of 5 μg of TSf seven days after completion of immunization schedule. Results are expressed as “delta mm” which was the difference between the values obtained after and before inoculation. **D.** Representative dot plots from CD8+ splenocytes from TSf-ISPA immunized and PBS-inoculated mice cultured in the presence of *T. cruzi* homogenate. **E.** Splenocytes from TSf-ISPA immunized mice showed higher production of IFN-γ within CD8+ cultured cells. **F.** Splenocytes from TSf-ISPA immunized mice showed an increase in the mean fluorescence intensity (MFI) of IFN-γ within CD8+ cultured cells. Data are expressed as means + standard deviations. Results shown are representative of 2-3 independent experiments (*n* = 4-10 mice per group), *p < 0.05, Mann-whitney test.

To first evaluate the utility of the TSf antigen, mice were immunized three times using ISPA as adjuvant and the generation of plasma specific antibodies anti-TSf (IgG1 and IgG2a) were assessed by ELISA. As shown in Figure 2A, TSf-ISPA immunized mice showed high titers of specific IgG2a antibodies and relatively low titers of IgG1 antibodies. In addition, a trypomasigote lysis assay was performed to evaluate the lytic functionality of the antibodies elicited. Serum from TSf-ISPA immunized mice showed lytic capacity as compared to PBS-inoculated mice (Figure 2B).

To analyze the influence of the vaccine candidate *in vivo*, footpad testing for DTH was performed. As shown in Figure 2C, TSf-ISPA immunized mice showed a marked increase in footpad thickness as compared with control groups (p < 0.05).

Finally, splenocytes were cultured with *T. cruzi* homogenate and IFN-γ production was assessed by flow cytometry. Higher percentage of intracellular IFN-γ was detected within CD8+ cells from TSf-ISPA immunized mice as compared with PBS-inoculated mice. In addition, higher mean fluorescence intensity (MFI) was detected in CD8+ cells from immunized mice (Figure 2D, 2E and 2F). On the other hand, a tendency but not significant increase in the percentage of IFN-γ was detected within the CD4+ compartment at the day analyzed, and a similar result was obtained concerning the MFI of IFN-γ in CD4+ cells from immunized mice (Supplementary Figure 1).

Taking together, the humoral and cellular immune parameters analyzed show that the TSf-ISPA formulation elicits an immune response that includes components of a Th1 response.

### TSf-ISPA immunization slightly affected the MDSC population

**Figure 3:**
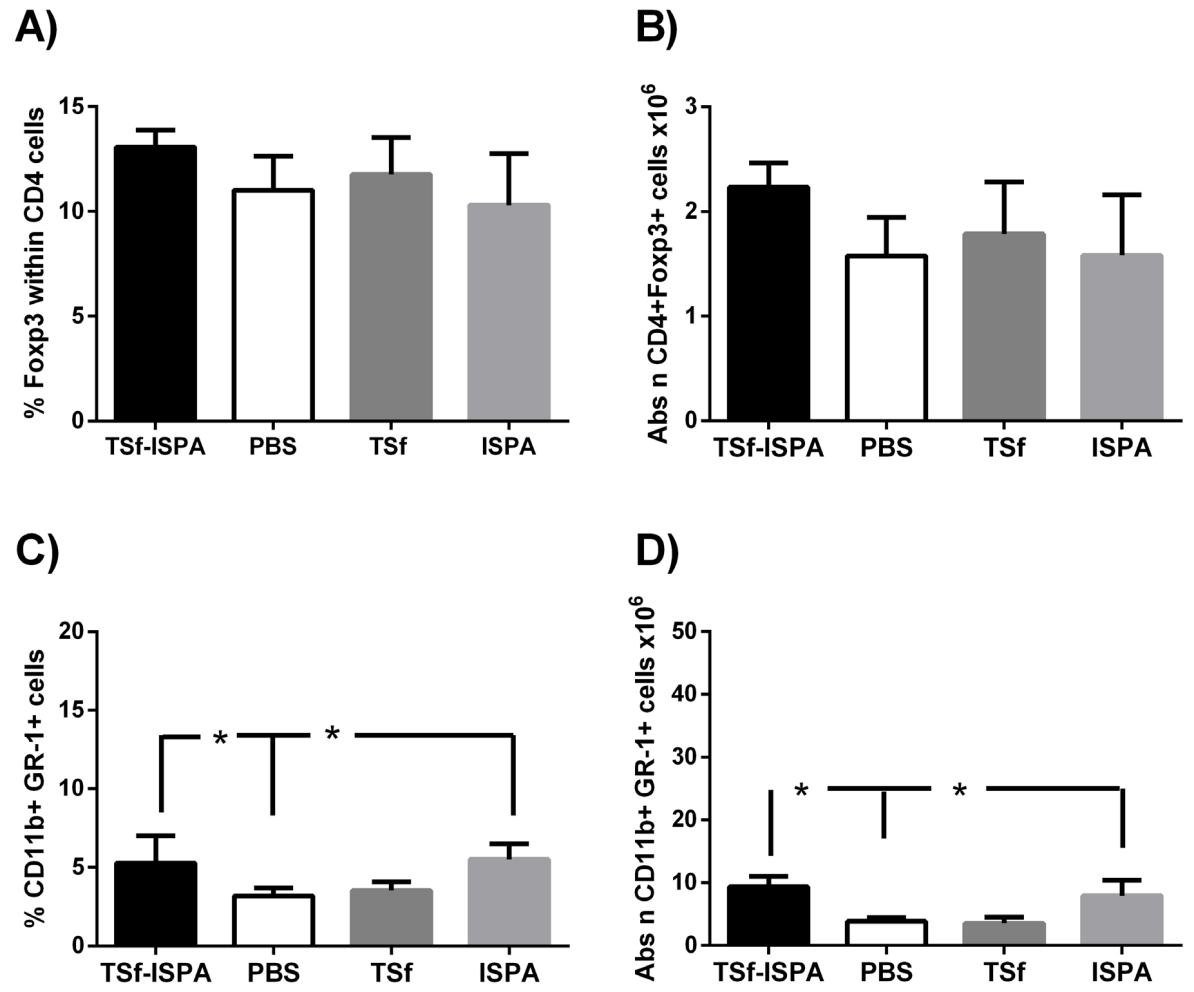
Changes in Foxp3+ CD4+ T cell populations and CD11b+ GR-1+ cells in the spleen of Balb/c mice, seven days after the last immunization **A.** FACS analysis of the expression of intracellular Foxp3+ within CD4+ cell in control (PBS-inoculated), TSf-alone, ISPA-alone and TSf-ISPA inoculated mice **B.** Absolute number of CD4+Foxp3+ cells (x10^6^) in the spleen of inoculated mice **C.** FACS analysis of the expression of CD11b+ GR-1+ MDSC cells in control (PBS-inoculated), TSf-alone, ISPA-alone and TSf-ISPA inoculated mice **D.** Absolute number of CD11b+ GR-1+ cells (x10^6^) in the spleen of inoculated mice. Data are expressed as means + standard deviations (*n* = 4 per group). The results are representative of two independent experiments, *p* < 0,05, Mann-whitney test.

Recent evidence suggests that vaccines may affect population of cells able to dampen the efficacy of immunization [24], [25], [26], [27]. Taking this issue into consideration, alterations in CD4+Tregs and MDSC cells were assessed one week after the last immunization. No significant differences were observed neither in Foxp3+ cells within CD4+ cells nor in the absolute number of CD4+Foxp3+ cells in the spleen of TSf-ISPA immunized mice as compared to ISPA-alone, TSf-alone or PBS-immunized mice (Figure 3A and 3B). On the other hand, a slight increase in the percentage and number of CD11b+ GR-1+ MDSC cells were observed in the spleen of TSf-ISPA-immunized and ISPA-immunized mice as compared to PBS-immunized mice (Figure 3C and 3D).

Taken together, results presented herein showed that TSf-ISPA immunization caused an important immune response that includes components of a Th1 profile, slightly influencing MDSC cells.

Of interest, no differences in the humoral or cellular immune response were registered when mice were immunized only with TSf antigen or ISPA adjuvant, excepting by a small increase in CD11b+ GR-1+ MDSC cells after ISPA adjuvant administration.

### TSf-ISPA immunized mice showed higher survival rates and decreased parasitemia

**Figure 4:**
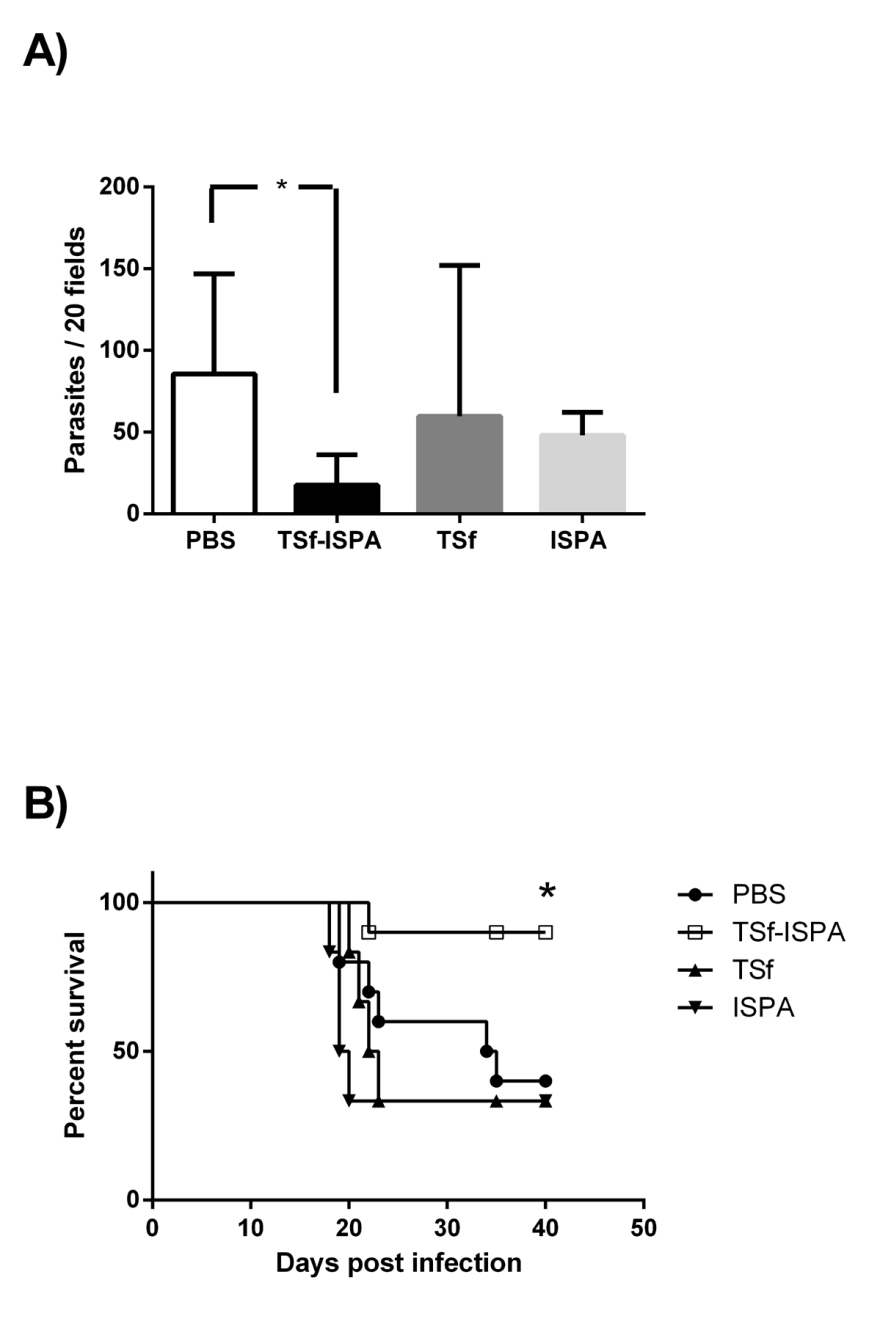
Parasitemia and survival rates in immunized mice after *T. cruzi* challenge Mice immunized with TSf-ISPA, TSf, ISPA and PBS were challenged with 1000 trypomastigotes of the Tulahuen strain 14 days after the last immunization. **A.** Parasitemia from PBS-inoculated (PBS, white bar), TSf-ISPA immunized (TSf-ISPA, black bar), TSf-immunized (dark gray bar) and ISPA-immunized (gray bar) mice at day 21 post infection. **B.** Survival rates are shown for TSf-ISPA immunized and infected mice (TSf-ISPA), PBS-inoculated and infected mice (PBS), TSf-immunized and infected mice (TSf), ISPA-immunized and infected mice (ISPA). The results are representative of two independent experiments (*n* = 6-10 mice per group), *p < 0,05, Mann-Whitney test was used for parasitemia analysis, Mantel-Cox Long rank test was used for survival analysis between TSf-ISPA vs PBS mice.

After the evaluation of the immune response elicited by the vaccine candidate itself, the protection efficacy of the TSf-ISPA formulation was evaluated during an *in vivo* challenge with 1000 trypomastigotes of Tulahuen *T. cruzi* strain. Of interest, TSf-ISPA immunized mice showed a lower number of blood parasites as compared to PBS-inoculated and infected mice, at day 21 post infection (Figure 4A). Moreover, the formulation was able to increase the survival from 40% in PBS-inoculated mice to 90% in TSf-ISPA-immunized mice (Figure 4B), indicating that the formulation under study was able to confer protection against a *T. cruzi* challenge. Although the administration of ISPA before *T. cruzi* infection decreased parasitemia at day 21 post infection, this result did not correlate with an increased survival, indicating that the treatment with the adjuvant alone does not confer protective capacity. Finally, the administration of TSf alone neither decreased parasitemia nor improved the survival of *T. cruzi* infected mice (Figure 4).

### After *T. cruzi* infection, IFN-γ expression increased in TSf-ISPA immunized and PBS-inoculated mice

**Figure 5:**
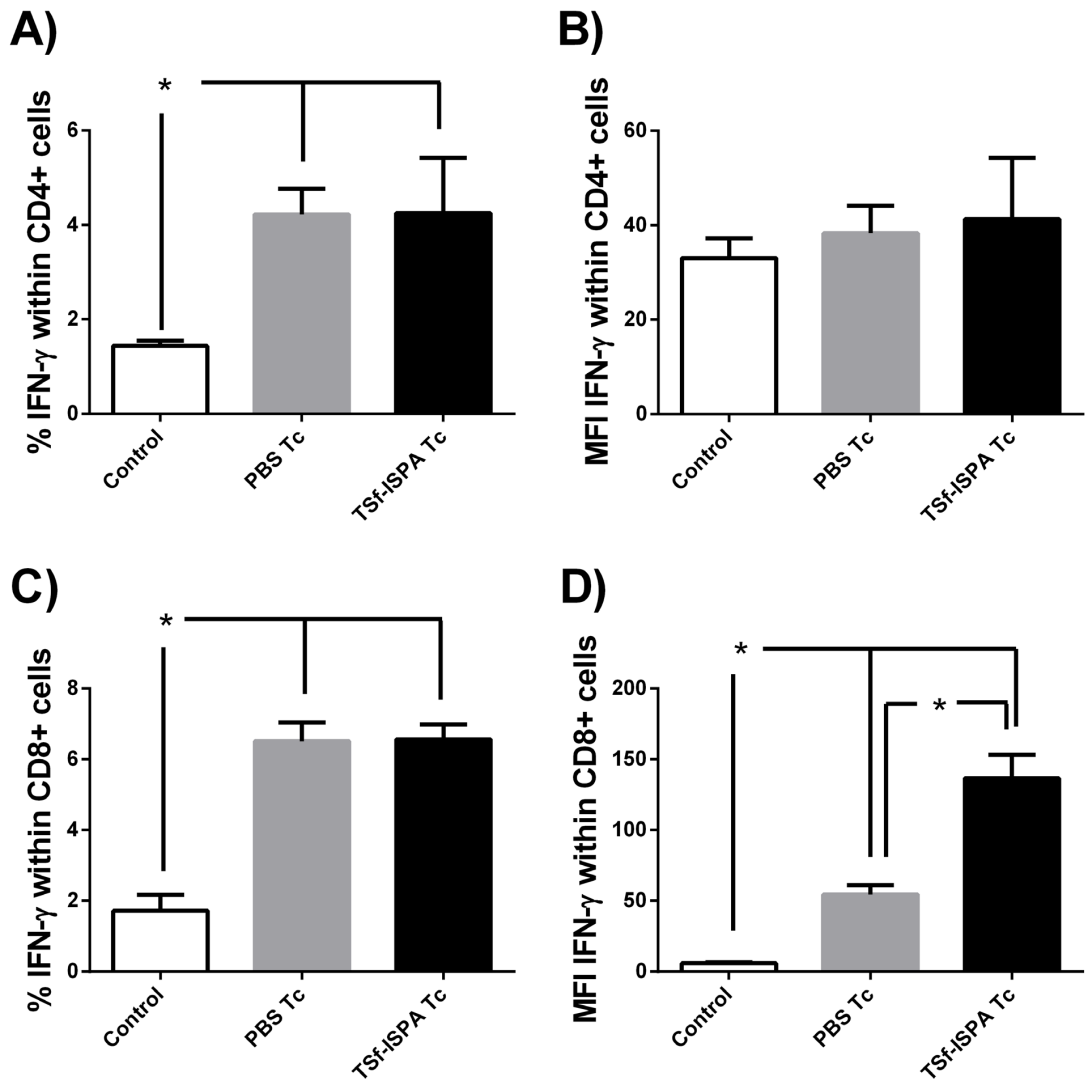
Production of IFN-γ within CD4+ and CD8+ *in vitro* Splenocytes from control non-infected (control), PBS-inoculated and infected mice (PBS Tc) and TSf-ISPA immunized and infected mice (TSf-ISPA Tc) were cultured with *T. cruzi* homogenate during 40h. PMA and monesin were added to the culture four hours before flow cytometry staining with anti-CD4, anti-CD8 and anti-IFN-γ antibodies. **A.** Percentage of IFN-γ production within splenic CD4+ T cells **B.** MFI of IFN-γ within CD4+ cells. **C.** Percentage of IFN-γ production within splenic CD8+ T cells, **D.** MFI of IFN-γ within CD8+ cells (*n* = 4/group). Data shown are representative of two independent experiments, *p < 0.05, Mann-whitney test.

The production of IFN-γ was assessed at day 21 post infection as a typical parameter of the effector immune response. As shown in Figure 5A and 5C, *T. cruzi* infection caused similar increases in IFN-γ production by CD4+ splenocytes from both PBS-inoculated and *T. cruzi* infected (PBS Tc) and TSf-ISPA immunized and *T. cruzi* infected mice (TSf-ISPA Tc), as compared to control non-infected mice (control). No significant difference was detected at day 21 of infection in MFI values of IFN-γ expression within CD4+ cells among the groups under study. On the other hand, infection caused similar and significant increases in the percentage of IFN-γ expression within cultured CD8+ cells from PBS Tc or TSf-ISPA Tc mice as compared to control non-infected mice. Strikingly, TSf-ISPA Tc mice showed a notable increase in MFI of IFN-γ expression in CD8+ cultured cells as compared to control and PBS Tc mice.

### After *T. cruzi* infection, TSf-ISPA formulation slightly increased Treg cells but notably decreased MDSCs cells

**Figure 6:**
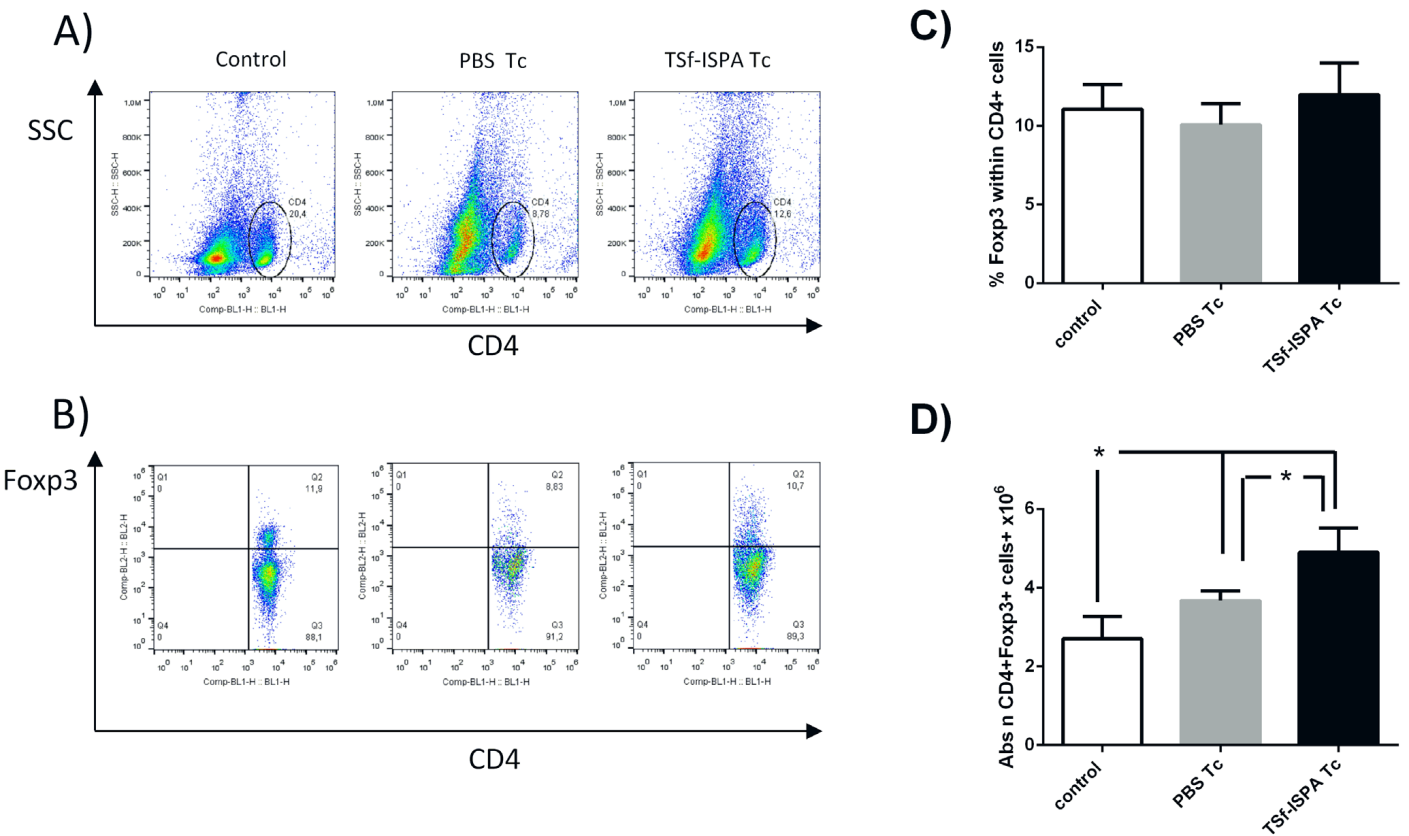
Changes in spleen CD4+ Foxp3+ cells 21 days post *T. cruzi* infection Expression of CD4 and intracellular Foxp3 was analyzed by FACS. **A.** the Figure shows representative dot plots used for selecting the CD4 region based on side-sideward scatter (SSC) in the Y-axis and CD4 expression in the X axis. **B.** Foxp3+ expression was assessed in the CD4 region in non-infected (control), PBS-inoculated and *T. cruzi* infected (PBS Tc), and TSf-ISPA immunized and *T. cruzi* infected mice (TSf-ISPA Tc). **C.** Percentage of Foxp3+ expression within CD4+ cells among groups **D.** Absolute number of CD4+Foxp3+ cells (10^6^). Data are expressed as means + standard deviations (*n* = 4 per group). *, *p* < 0.05, Mann-whitney test. Data shown are representative of two independent experiments.

Although several reports have addressed the involvement of CD4 Foxp3+ Treg cells during the acute phase of *T. cruzi* infection, the role of this population remains unclear [32], [33], [34], [35]. To analyze whether the TSf-based immunogen affected this immunomodulatory population after a challenge with *T. cruzi*, flow cytometry was performed at day 21 after infection. No significant difference was detected concerning the percentage of Foxp3+ Treg cells within CD4+ cells in the spleen of non-infected (control), PBS-inoculated and infected (PBS Tc) and TSf-ISPA inoculated and infected mice (TSf-ISPA Tc) (Figure 6A, 6B and 6C). However, the absolute number of CD4+Foxp3+ cells was significantly increased in the spleens from PBS Tc and TSf-ISPA Tc mice, correlating with the total cellularity of the spleens after *T. cruzi* infection. Moreover, TSf-ISPA Tc mice showed a significant increase of Treg cell number as compared to PBS Tc mice.

**Figure 7:**
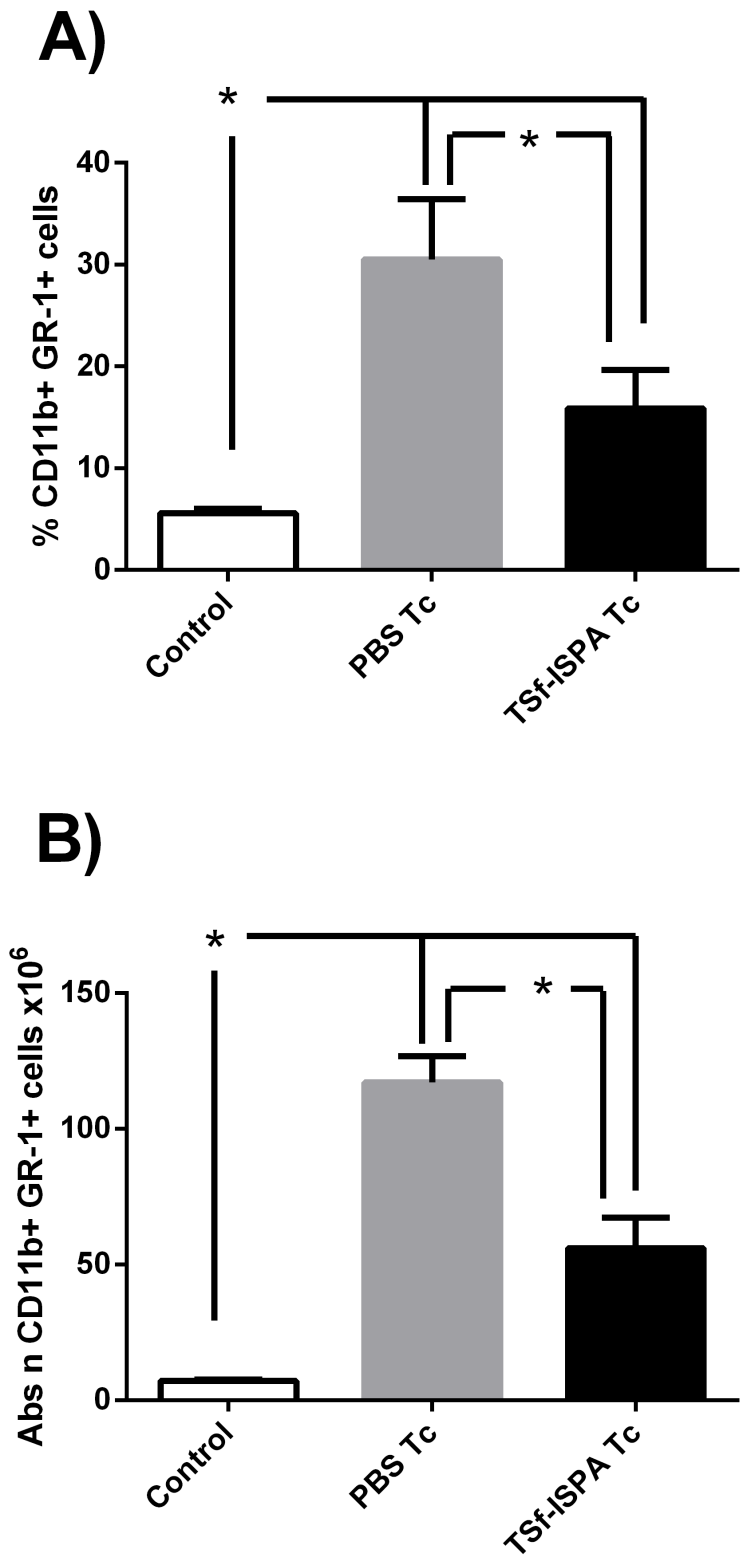
Changes in spleen CD11b+ GR-1+ cells at day 21 post *T. cruzi* infection Expression of CD11b+ and GR-1+ was analyzed by FACS in control non-infected (control), PBS-inoculated and infected mice (PBS Tc) and TSf-ISPA immunized and infected mice (TSf-ISPA Tc). **A.** percentage of CD11b+ GR-1+ cells. **B.** Absolute number of CD11b+ GR-1+ cells (x10^6^). Data are expressed as means + standard deviations (*n* = 4 per group). *, *p* < 0.05, Mann-whitney test. Data shown are representative of two independent experiments.

It has been reported that *T cruzi* infection induces an important increase of MDSCs in the spleen [31], [30]. In line with previous reports, infected mice showed a very important increase in the percentage and number of splenic CD11b+GR-1+ MDSCs. However, TSf-ISPA immunized and infected mice showed a marked decrease in the percentage and absolute number of MDSC as compared to PBS-inoculated and infected mice (Figure 7A and 7B), strongly suggesting that the formulation exerts an important effect on this population of suppressor cells.

Of note, at day 14 post infection, no differences were observed neither in Treg cells nor MDSC cells between TSf-ISPA-immunized and PBS-immunized mice (data not shown).

**Figure 8:**
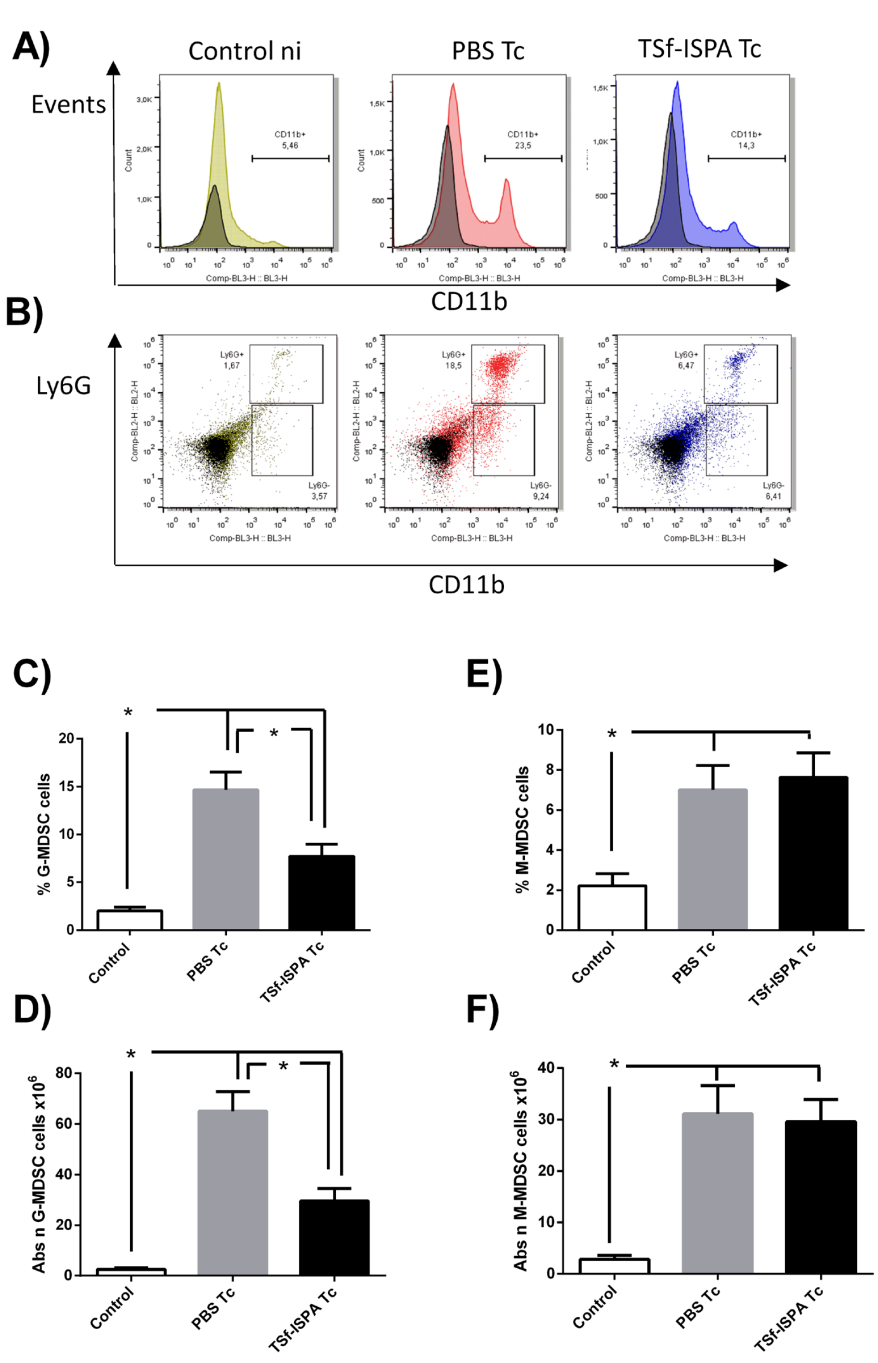
Changes in spleen CD11b+ Ly6G+/- cells at day 21 post *T. cruzi* infection Splenocyte suspensions were prepared and expression of Ly6C-FITC, Ly6G-PE and CD11b-PerCP-Cy5.5 expression was analyzed by flow cytometry in control non-infected mice (control), PBS-inoculated and infected mice (PBS Tc) and TSf-ISPA immunized and infected mice (TSf-ISPA Tc). **A.** Representative dot plots showing alterations in the percentage of spleen CD11b+ cells among groups. **B.** Representative dot plots showing alterations in the percentage of CD11b+Ly6G+ (G-MDSC) and CD11b+Ly6G- (M-MDSC) cells among groups. **C.** Percentage of G-MDSC CD11b+Ly6G+ cells, **D.** Absolute number of G-MDSC CD11b+Ly6G+ cells (x10^6^). **E.** Percentage of M-MDSC CD11b+ Ly6G- cells. **F.** Absolute number of M-MDSC CD11b+Ly6G- cells (x10^6^). Data are expressed as means + standard deviations (*n* = 4 per group). *, *p* < 0.05, Mann-whitney test. Data shown are representative of three independent experiments.

Two subsets of MDSC have been defined according to the expression of Ly6G [39]: granulocytic MDSC (G-MDSC) CD11b+ Ly6G+ and monocytic MDSC (M-MDSC) CD11b+ Ly6G-. Thus, flow cytometry was carried out to evaluate whether one or both of these populations were affected by the formulation after *T. cruzi* challenge. Of interest, TSf-ISPA immunized and infected mice only showed a significant decrease in the percentage and absolute number of G-MDSC population as compared to PBS-inoculated and infected mice (Figure 8A, 8B, 8C, 8D, 8E, 8F).

In addition, to better characterize the MDSC population, Ly6C expression was analyzed within Ly6G+/- populations. In our hands, both MDSC populations showed Ly6C expression (Supplementary Figure 2).

Taking together, our results strongly suggest that TSf-ISPA formulation mainly influenced the G-MDSC population (CD11b+Ly6G+Ly6C+) after *T. cruzi* infection, without severely affecting the M-MDSC population (CD11b+Ly6G-Ly6C+).

### Hearts from chronically infected mice that were immunized with TSf-ISPA did not show a significant increase of fibrosis

**Figure 9:**
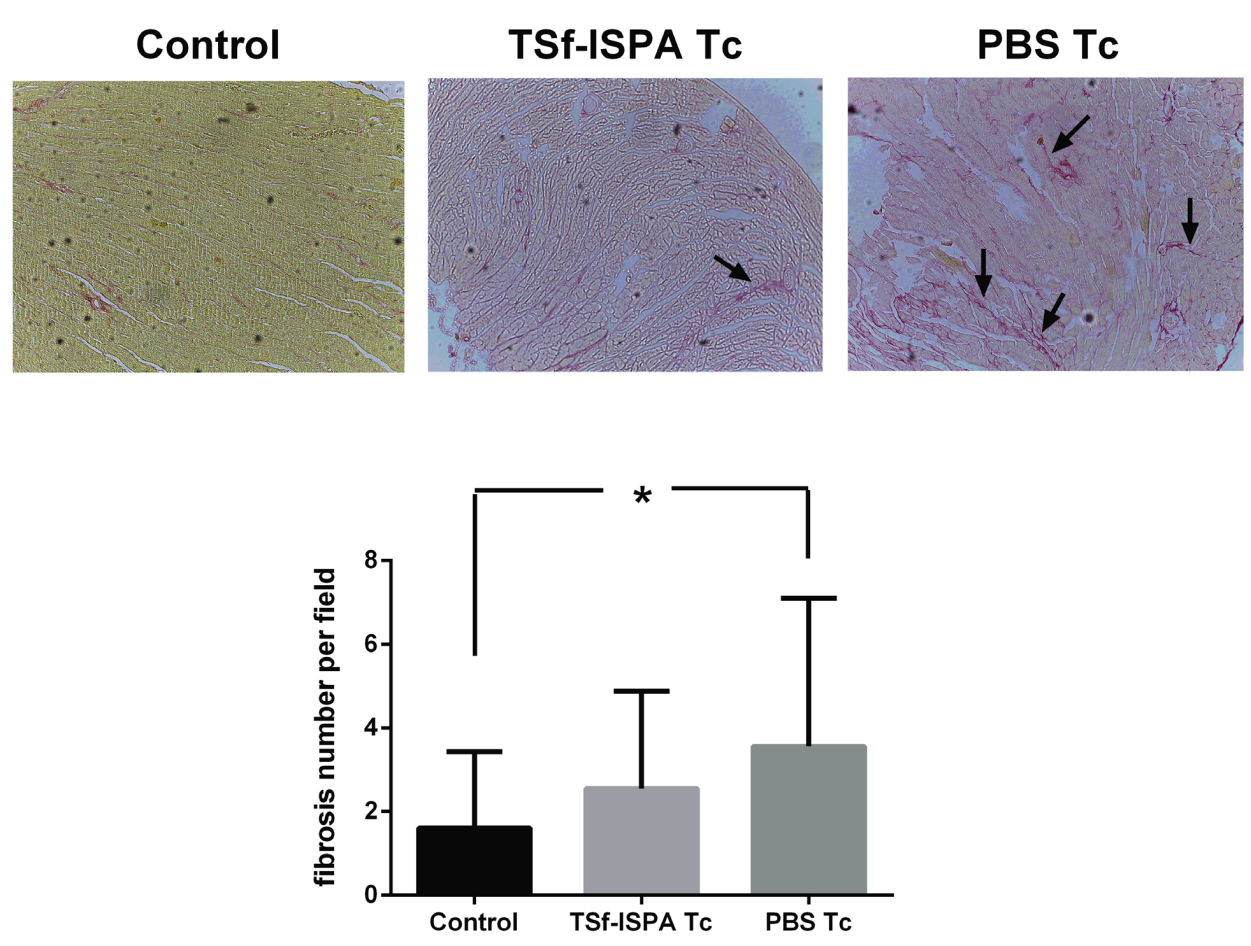
Tissue pathology Picrosirius red staining of hearts after five months of infection. Arrows indicate examples of red staining considered as collagen fibers. Magnification 100x. (*n* = 3-4 mice analyzed per group). *p < 0.05. (For interpretation of the references to color in this figure legend, the reader is referred to the web version of this article).

Histological analysis of hearts from chronically infected mice was performed five months after the challenge. Hearts from PBS-treated and infected mice showed an extensive and significant increase of fibrosis as compared to control non-infected mice. In contrast, hearts from immunized and infected mice only exhibited a slight and non-significant increase of fibrosis as compared to control non-infected mice (Figure 9). Taken together, our results support that the TSf-ISPA formulation modulates both arms of the immune response in such a way that increases the survival against a challenge and ameliorates the outcome of the infection.

## DISCUSSION

Much progress has been made in the field of *T. cruzi* vaccine development. In this regard, the parasite antigens and their characteristics have been widely described; adjuvants needed to elicit a beneficial immune response are more available, and the components of the immune systems that influence the outcome of the infection are better understood. Thus, new formulations can be readily prepared and assayed to advance toward an immunotherapeutic treatment of the disease. Here we report the cloning, purification and assessment of a new TS-based vaccine candidate that was able to protect against *T. cruzi* infection by affecting both the effector and the suppressor arm of the immune response.

Based on the available knowledge regarding the trans-sialidase protein from *T. cruzi*, a new antigen of 290 aminoacids was obtained. A fragment size that included less than 1000 bp was selected considering a future potential expression in recombinant bacteria designed to be used as delivery system. Two additional issues considered for the design were the facts that an active TS may be harmful to the host and that the SAPA domain is immunodominant but generates irrelevant antibodies that delays the production of antibodies against the active site of the enzyme [40], [15], [41]. To circumvent these constraints, we sought to develop a recombinant protein that lacked the SAPA domain and the Nh2 terminal region, which would render inactive the protein because such a fragment would not be able to bind galactose [42].

The TSf fragment obtained after PCR amplification, cloning and expression in *E. coli* showed 90% aminoacidic identity against a TS sequence reported in the GenBank AJ276679 that has protective capacity against *T. cruzi* [15]. In addition, the TS sequence obtained showed as much as 92% identity against all the indexed sequences in the GenBank from different *T. cruzi* strains. These high identity percentages not only suggest that the cloned aminoacid secuence belongs to the TS family, but also raise the possibility that it could be capable to protect against several *T. cruzi* strains. There is only one previous study assessing identity and protection correlation [51]. In that work, 85% of identity between proteins of the TS family from different *T. cruzi* strains was enough to generate protection when used as immunogen against a lethal challenge. Studies assessing the protective capacity of TSf against other *T. cruzi* strains are actually in progress.

TSf was formulated with the ISPA adjuvant, consisting of lipidic cages developed in our own laboratory (manuscript in preparation), with the aim of redirecting the immune response to a profile that included several components of a Th1 response, an immunological requirement that has been shown by several groups [19], [20], [21], [22]. In line with our unpublish data, the TSf-ISPA formulation elicited a potent immune response that included components of a Th1 profile, as supported by several immunological parameters. TSf-ISPA immunization elicited specific IgG2a and IgG1 plasma antibodies against TSf with a marked ratio IgG2a/IgG1>1 and trypomastigote lytic activity. In addition, DTH analysis showed that TSf-ISPA immunized mice had a remarkable increase in footpad inflammation, correlating with the development of a measurable *in vivo* cellular immune response. Finally, the percentage as well as the MFI of IFN-ɣ were increased in CD8+ splenocytes from TSf-ISPA immunized mice as compared to splenocytes from PBS-inoculated mice.

It has been reported that immunization with the TS protein alone inhibits a Th1 response [23]. Results presented herein strongly suggest that ISPA adjuvant influences the immune response, eliciting several components of a Th1 profile. In addition, the IgG2a/IgG1 ratio, magnitude of DTH and IFN-ɣ expression were similar to a previous study conducted by our group using another inactivated-TS antigen expressed in yeast and formulated with ISCOMATRIX [11]. Both reports are in line and support that inactive forms of the TS protein may be promising candidates to be used in vaccine protocols.

Currently it is well accepted that the immune system is composed not only by an effector arm but also by an immunomodulatory counterpart. Despite this fact, few works based on vaccine development analyze whether immunization affects populations of cells with regulatory/suppressor capacity during vaccination or challenge with a pathogen [24]. In this work, two of the best characterized immunomodulatory populations were studied, CD4+ Foxp3+ Treg cells and MDSC cells. Of interest, TSf alone, ISPA adjuvant or TSf-ISPA formulation did not induce alterations neither in the percentage of Foxp3+ cells within CD4+ nor in the absolute number of CD4+Foxp3+ Treg cells in the spleen, suggesting that the TSf-ISPA formulation may have an important advantage, because it does not induce significant increases of CD4+ Foxp3+ cells at the stage of immunization, as has been shown in other reports [25], [27], [26]. In addition, the adjuvant alone and the TSf-ISPA formulation induced slight increases in the percentage and absolute number of MDSC CD11b+ GR-1+ cells in the spleen. An increase of myeloid-derived suppressor cells has been reported after vaccination with Salmonella or mycobacterium bovis bacillus Calmette-Guerin [43], [44], suggesting that measurement of the MDSC population may be valuable after an immunization protocol. Taking together, our results suggest that the TSf-ISPA formulation may represent a valuable tool for vaccine design as it induced an immune response comprising components of a Th1 profile, did not elicit relevant increases of Treg cells and only induced slight increases of MDSC cells.

Fifteen days after the last immunization, mice were challenged with 1000 trypomastigotes Tulahuen in order to assess the performance of the vaccine candidate during an *in vivo* challenge. Of interest, TSf-ISPA immunization increased the survival from 40% in control mice to 90% in immunized mice, indicating that the formulation had an important protective capacity, very similar to previous studies using other formulations based on recombinant TS-antigens [15], [11]. Consistent with the increased survival, TSf-ISPA immunized mice showed decreased levels of blood parasites along the infection. In effect, the level of parasites observed in immunized mice was notably decreased was notably decreased as compared to PBS-inoculated mice, suggesting that TSf-ISPA immunized mice were better prepared to cope the infection.

IFN-ɣ expression was measured as a typical parameter of an ongoing immune effector response. Infection similarly increased the percentage of IFN-ɣ expression in cultured CD4+ and CD8+ splenocytes from PBS-inoculated and TSf-ISPA immunized mice as compared to splenocytes from non-infected mice. Of interest, MFI of IFN-ɣ expression was highly increased in CD8+ splenocytes from TSf-ISPA immunized and infected mice, raising the possibility that this population could be involved in the better outcome observed in immunized mice. Further studies will be conducted to get deep into the immune effector mechanisms by which the TSf-ISPA formulation confer protective capacity.

Concerning the regulatory/suppressor arm of the immune response, alterations in CD4 Foxp3+ regulatory T cells and MDSC cells were also analyzed. Although several reports have assessed the existence of alterations of CD4+ Foxp3+ regulatory T cells during *T. cruzi* infection, the role of this population during the acute phase of infection still remains unclear. Some studies have suggested that Treg cells do not play a relevant role during *T. cruzi* infection, as depletion of this population did not influenced mice survival [32], [33], [34]. However, a recent report has shown that Treg cell induction with dexamethasone and IL-2 is able to significantly increase mice survival after a lethal challenge [35]. In our hands, we found a significant increase in the absolute number of CD4+ Treg splenocytes in PBS-inoculated and TSf-ISPA immunized and infected mice as compared to non-infected mice. Moreover, TSf-ISPA immunized and infected mice showed a higher number of Tregs as compared to PBS-inoculated mice. Considering all the reports about Treg cells, it seems that this population can play a role during *T cruzi* infection, at least when Tregs cells are induced, as has been previously shown [35]. Thus, the possibility exists that the increase in the number of Treg cells observed in our model play some beneficial role in the increased survival registered in TSf-ISPA immunized mice. On the other hand, some reports have shown that the presence of Foxp3+ Treg cells correlates with a better outcome of the chronic phase of the infection. In this sense, it has been shown that patients in an indeterminate state of infection possess a higher number of Foxp3+ regulatory cells as compared with symptomatic patients [36], [37]. Taking together, the evidence suggests that Foxp3+ Treg cells could play a beneficial role during the acute phase of infection and might coexist with a better outcome of the infection during the chronic stage. Thus, an immunogen that does not induce significant increases of Treg cells during the immunization process but elicits an increase of that population after *T. cruzi* infection would be valuable in the design of a prophylactic as well as a therapeutic formulation, perhaps contributing to avoid the transition from the indeterminate phase to the symptomatic stage of the infection.

Regarding MDSC cells, marked increases were detected in this suppressor population after the challenge of *T. cruzi* in PBS-inoculated mice, a fact that correlates with previous reports [30], [31]. However, it is to note that a marked decrease in MDSC cells was observed in TSf-ISPA immunized mice as compared to control infected mice.

This decrease was mainly due to a decrease in the granulocytic MDSC (CD11b+ Ly6G+ Ly6C+) while no significant difference was observed concerning the monocytic MDSC (CD11b+ Ly6G- Ly6C+). Very strikingly, a recent report has shown that MDSC depletion highly decreases mice survival, strongly suggesting that this population plays an important role during the acute phase of infection [30]. The fact that TSf-ISPA immunized mice showed a higher survival and decreased parasitemia in spite of having decreased levels of MDSC suggests that the formulation may favorably modulate the immune response to *T. cruzi* without the need of a generalized immunosuppression involving MDSC cells. In this sense, it has been reported that a vaccine based on a specific strain of Salmonella is able to confer protection against other strains precisely by decreasing the generalized immunosuppression caused by MDSC cells [43]. Thus, in our model, the possibility exists that the TSf-ISPA formulation generates a better control of the infection by modulating the immune response in a way that increases components of a Th1 response, increases the absolute number of specific CD4+Foxp3+ Treg cells with protective capacity, and decreases the generalized immunosuppression induced by MDSC cells. Moreover, this favorable effect of the TSf-ISPA formulation seems to also influence beneficially the outcome of the pathology, since hearts from immunized mice did not showed a significant increase of fibrosis at the chronic phase of infection.

Some recent reports have described that MDSC may expand or inhibit Treg cells, depending on the model [45], [46]. The cross-talk between these immunomodulatory populations has only begun to be elucidated. In this sense, our model might constitute a valuable tool to analyze putative interactions since a decrease in the number of MDSC was accompanied by increases in the number of Treg cells.

It is well-known that TS activity mediates several biological effects leading to the subversion of host immune system (reviewed in [47]). If TS influences MDSC increases by an unknown interaction, anti-TS antibodies may account for the lower increases of MDSC observed in TSf-immunized mice. Putative effects of TS on MDSCs could be related to the output from the bone marrow or their maturation state in the periphery. In any case, it would not be a minor event since the increase in this population in the spleen constitutes a huge and biologically relevant alteration during *T. cruzi* infection, as evidenced by the fact that depletion of MDSCs severely affects mice survival.

Recent studies have focalized in the importance of MHCI T-cell epitopes from TS regarding their immunodominance and their protective capacity. In particular, it has been postulated that the sequences of CD4+ T cell epitope KVTERVVHSFRLPALVNV (p7) and CD8+ T-cell epitope IYNVGQVSI (TSKd1) have a pivotal role to obtain protection against *T. cruzi* infection in BALB/c mice [48]. Since the TSf fragment does not include neither the p7 nor the TSKd1 epitope but showed protection capacity against *T. cruzi* infection, our results suggest that the p7 and the TSKd1 epitope are not absolutely relevant for *T. cruzi* protection in BALB/c mice. It remains to be studied whether the p7 and TSKd1 may increase the protection capacity and currently another TS fragment containing the Nh2-terminal region of the TS is been cloned by our group.

Herein we have described the development of a new TS-based formulation able to protect against *T. cruzi* challenge by modulating the effector as well as the immunomodulatory arm of the immune system. To our knowledge this is the first report showing that a subunit vaccine candidate may affect MDSC cells after the challenge with a pathogen and further research will be conducted to elucidate the mechanisms that underlie this striking event. The present work may highlight the relevance of studying both arms of the immune system when a vaccine candidate is to be rationally tested.

## MATERIALS AND METHODS

### Gene amplification, expression, and purification of the TSf recombinant antigen

The GenBank sequence AJ276679 was used to design primers to amplify by PCR a TS fragment of reduced size (TSf) [15]. Restriction sites were added to the primers ends; Forward: 5´-GAATTCGGTGATGAAAATTCCGCCTA-3´ (*Eco* R1 site underlined) and reverse: 5´-GAGCTCAGGTCCTGGCTCAAGAACAA-3´ (Sac 1 site underlined). Genomic DNA from *T. cruzi* CL-Brenner was used. The PCR program consisted of denaturation at 94°C for 30 s, annealing at 54°C the first ten cycles and 62°C the remaining 20 cycles for 30 s, and extension at 72°C for 90 s (in a total of 30 cycles) in 50 μL of PCR buffer containing 0,5 units of Go Taq^®^ polymerase (Promega, USA), 10 μL of Go Taq^®^ buffer 5X (Promega, USA), 0.2 μM of each primer (Invitrogen, Argentina), 1.5 mM of MgCl_2_, 0.2 mM of each DNTPs and 100 ng of genomic DNA. PCR products were evaluated on a 1% agarose gel under UV light in the presence of GelRed^TM^ (Biotium, USA). The desired gene fragments were excised and purified by gel extraction kit (Promega, USA). The amplified sequence was cloned into the pGEM^®^-T easy vector (Promega, USA) using a T4 DNA Ligase enzyme (Promega, USA) at 4°C overnight. Then, the plasmids were transformed in *E. coli* DH5α competent cells as previously described [49] and mini preparations of pGEM°-T easy vector were obtained using Miniprep isolation columns (QIAGEN, Valencia, CA), following the manufacturer's instructions. After sequencing the plasmids, the identity of the PCR products was verified by alignment with all the TS genes indexed in the GenBank and the particular sequence (AJ276679) that was used to design the primers. The SIM- Alignment Tool for protein sequences was used for alignments.

The TSf sequence was subcloned into pET-28a vector (Novagen^TM^, Germany) in the *ECO* R1 and *SAC* 1 restriction sites. *E. coli* BL21 (DE3) competent cells were transformed with the recombinant plasmids. One mL of overnight LB culture was transferred into 100 mL of LB medium containing 50 μg/mL kanamycin and was allow to grow in a shaking incubator at 37°C for 3 h. IPTG was added in a final concentration of 0,5 mM and the induction was carried out at room temperature in a shaking incubator for 16 h.

After induction, the cultures were centrifuged at 5000 rpm and the pellets were rinsed three times with triton 1%. After dilution with PBS, bacteria were sonicated and inclusion bodies containing TSf protein were solubilized in 8 M urea, 50 mM Tris/HCl, 5 mM EDTA (pH 8) buffer. The presence of the recombinant fragment was detected by SDS-PAGE (15% polyacrylamide gels).

Due to the presence of His-tag, provided by the expression vector, the TSf was purified by Ni^2+^-affinity chromatography (Invitrogen^TM^, USA), using a gradient of buffer solutions of urea 8M-imidazole 20mM to urea 8M-imidazole 500mM, following the manufacturer's instructions. The eluted fractions containing the TSf protein were dialyzed against 0,5 M urea, 50 mM Tris/HCl, 5 mM EDTA (pH 8.5) buffer. Finally, an electrophoresis analysis SDS-PAGE was performed to verify the purity of the TSf antigen and the bicinchoninic method was used to quantify protein concentration as previously described [50]. A TSf batch of nearly 10 mg was prepared. Endotoxin in the recombinant proteins was measured by the Limulus amebocyte lysate (LAL) assay kit (Genscript, USA Inc); the levels were < 10 endotoxin units (EU)/mg protein.

### Mice

BALB/c female mice (6-8 weeks old) used in all experiment procedures were obtained from the Centro de Medicina Comparada, ICIVET-CONICET UNL, Argentina. All protocols for animal studies were approved by the Animal Care & Use Committee, according to the Institutional guidelines.

### Immunization schedules, infection protocol, parasitemia and survival

BALB/c mice (*n* = 6-10/group) were immunized with three subcutaneous doses, one every two weeks, containing 10 μg of TSf with 26 μg of ISPA as adjuvant. ISPA adjuvant is composed of liposomes with cage-like structures of 73,0 ±1,5, nm as assessed by dynamic light scattering. The components of the particles are phospatidilcholine (DPPC), cholesterol (CHOL), sterylamine (STEA), tocopherol (TOCOP) and saponin. ISPA was prepared in two steps. First, liposomes were prepared with the following final proportions: DPPC: 0,320% (4,35 mM), CHOL: 0.143% (3.70 mM), STEA: 0.0216% (0.8 mM), and TOCOP: 0.00074% (0.017 mM). Then, the suspension was extruded with a 50 nm membrane pore and a saponin solution in Acetate Buffer was added to liposomes (6.5mg/300ul for each ml of liposomes). Control groups were immunized with TSf alone, ISPA alone or PBS solution, following the same protocol. For infection, mice were challenged intra peritoneally with 1000 bloodstream trypomastigotes of Tulahuen strain, fifteen days after the last immunization. Parasitemia was monitored at days 14, 21 and 28 post-infection by examining 5 μl of blood by direct microscopic examination, as previously described [11]. Survival was recorded daily until day 100 post-infection.

### IgG subclass profile and titer determination

Blood was collected on day 7 after the third immunization dose to analyze the specific anti-TSf antibody production by ELISA. TSf (0.5μg) in carbonate-bicarbonate buffer (0.05 M; pH 9.6) was used to coat microtiter plates (Greiner Bio One). After ON incubation at 4°C, wells were rinsed with PBS-Tween20 0,05% and blocked with PBS/5% bovine serum albumin (BSA). Half-serial dilutions in PBS/1% BSA of serum samples were incubated by duplicate in TS-coated wells. Plasma specific antibodies against TSf were detected by incubation with IgG1 and IgG2a (1:10,000, Southern Biotechnology). Samples were read at 450 nm in an ELISA reader (Bio-Tek Instruments) after incubation with 50 μl of ready to use trimethylbenzidine (Invitrogen). The titer for each TS-specific Ig was defined as the end-point dilution that yielded an optical density (O.D.) higher than the 1/100 preimmune plasma dilution.

### Trypanolytic activity assay

Tulahuen trypomastigotes were purified from cultures of infected Vero cells. (5×10^4^) trypomastigotes were incubated for 6 h at 37°C with each serum sample (1/20 diluted) and a fresh human serum as a source of complement in a final volume of 50 uL RPMI 1640 (Gibco). The number of live trypomastigotes were determined using a Neubauer chamber. Lysis of 0% was determined based on the number of parasites incubated only with RPMI.

### Delayed-type hypersensitivity

DTH test was performed by intradermal challenge with 5 μg of TSf in the right hind footpad seven days after completion of immunization schedule. Hind footpad thickness was measured before antigen injection and after 48 h with a Vernier caliper (Stronger). Results were expressed as the increment in millimeters of footpad thickness induced by inoculation.

### Spleen cell culture and IFN-γ determination

Groups of mice were immunized with TSf-ISPA or PBS as described above. Fifteen days after the end of the immunization protocol, mice were sacrificed to analyze ex vivo IFN-γ production of splenocytes stimulated with *T. cruzi* homogenate. Briefly, spleens were aseptically harvested and homogenized. Red blood cells were eliminated and splenocytes were re-suspended in RPMI 1640 medium (Gibco) supplemented with 10% fetal bovine serum, 2%penicillin (100 μg/mL), streptomycin (100 U/mL) and 0.4 mM 2-mercaptoethanol.

Cells (1×10^6^)/mL/well were cultured in 24 or 48-well plates (Nunc) in supplemented RPMI. Splenocytes were stimulated with *T. cruzi* homogenate or RPMI as control. Concanavaline A (2.5-μg/mL) were used as positive control of stimulation. After 36 hours at 37**°** C and 5% CO_2_, cells were incubated with 75 ng/mL of 12-myristate 13-acetate (PMA, Sigma-Aldrich) and monensin (BD-Pharmingen) according to the manufacturer´s instructions. Four hours later, cells were washed twice with PBS, incubated with anti-Fc III/II receptor antibody for 30 min and stained with anti-CD4 (clone RM4-5) and anti-CD8 PerCP Cy5.5 during 30 min. Then, cells were washed and stained for IFN-γ using the IFN-γ kit for intracellular staining from BD- Phamingen according to the manufacturer´s instruction. Samples were acquired on an Attune NxT cytometer (Invitrogen) and analyses were performed using FlowJo software.

Additionally, mice were immunized and then infected with *T. cruzi*. In this case, mice were sacrificed 21 days after infection in order to analyze ex vivo IFN-γ production as described above.

### Flow cytometry of Treg cells and MDSC

The CD4+Foxp3+ (Treg cells) and MDSC were measured by flow cytometry at day 7 post immunization protocol and at day 21 post infection. Briefly, mice were sacrificed and spleen single cell suspensions were prepared by homogenization through a stainless steel mesh using PBS 3% fetal bovine serum (FBS). Spleen absolute numbers were determined by counting cells in Neubauer chamber using Turk solution and then red blood cells were eliminated by lysis with H_2_0 during 30 seconds. Finally, spleen cells were homogenized in PBS 3% FBS and 1.10^6^ cells were stained with the following antibodies from BD-Pharmingen: fluorescein isothiocyanate (FITC) anti-CD4 (clone RM4-5), FITC anti-Ly6C (clone AL-21), phycoeritrin (PE) anti-Ly6G (clone 1A8), PE anti-GR1 (clone RB6-8C5) and PerCP-Cy 5.5 anti-CD11b (clone M1/70). Cells marked with CD4 were also intracellular stained for PE anti-Foxp3, using mAb from Miltenyi Biotec, according to manufacturer´s instructions.

Samples were acquired on an Attune NxT cytometer (Invitrogen) and analyses were performed using FlowJo software.

### Histopathology

Hearts from immunized and *T. cruzi* infected mice were removed five months after the challenge. Each heart was transversally sectioned in two pieces and fixed in buffered formalin, (*n* = 3-4 mice per group). Then, paraffin-embedded 5um sections were subjected to picrosirius red staining as previously reported [11]. The number of fibrosis per field were quantified in a blind manner. Ten fields were assessed by heart.

### Statistical analyses

Multiple groups were first analyzed using nonparametric tests (Kruskall-Wallis test for k samples) and then Mann-whitney test was employed to analyze differences between two particular groups. Mantel-Cox Long rank test was used to evaluate survival curves. All analyses were performed using GraphPad Prisma 6.0 software (GraphPad, California, USA). Significance is indicated with (*) when p < 0.05 between the indicated groups.

## SUPPLEMENTARY MATERIALS FIGURES



## Abbreviations

*T. cruzi*: *Trypanosoma cruzi*
TS: Trans-sialidase
TSf: Trans-sialidase fragment
Treg: Foxp3+ regulatory T cells
MDSC: Myeloid-derived suppressor cell

## References

[1] World Health Organization (2015). Chagas disease in Latin America: an epidemiological update based on 2010 estimates. Wkly Epidemiol Rec.

[2] Schmunis GA (2007). Epidemiology of Chagas disease in non-endemic countries: the role of international migration. Mem Inst Oswaldo Cruz.

[3] Viotti R, Vigliano C, Armenti H, Segura E (1994). Treatment of chronic Chagas’ disease with benznidazole: clinical and serologic evolution of patients with long-term follow-up. Am Heart J.

[4] Dumonteil E, Bottazzi ME, Zhan B, Heffernan MJ, Jones K, Valenzuela JG, Kamhawi S, Ortega J, Rosales SP, Lee BY, Bacon KM, Fleischer B, Slingsby BT (2012). Accelerating the development of a therapeutic vaccine for human Chagas disease: rationale and prospects. Expert Rev Vaccines.

[5] Morillo CA, Marin-Neto JA, Avezum A, Sosa-Estani S, Rassi A, Rosas F, Villena E, Quiroz R, Bonilla R, Britto C, Guhl F, Velazquez E, Bonilla L (2015). Randomized Trial of Benznidazole for Chronic Chagas’ Cardiomyopathy. N Engl J Med.

[6] Gupta S, Garg NJ (2015). A Two-Component DNA-Prime/Protein-Boost Vaccination Strategy for Eliciting Long-Term, Protective T Cell Immunity against Trypanosoma cruzi. PLoS Pathog.

[7] Eickhoff CS, Giddings OK, Yoshida N, Hoft DF (2010). Immune responses to gp82 provide protection against mucosal Trypanosoma cruzi infection. Mem Inst Oswaldo Cruz.

[8] Cazorla SI, Frank FM, Malchiodi EL (2009). Vaccination approaches against Trypanosoma cruzi infection. Expert Rev Vaccines.

[9] Quijano-Hernandez I, Dumonteil E (2011). Advances and challenges towards a vaccine against Chagas disease. Hum Vaccin.

[10] Cerny N, Sanchez Alberti A, Bivona AE, De Marzi MC, Frank FM, Cazorla SI, Malchiodi EL (2016). Coadministration of cruzipain and GM-CSF DNAs, a new immunotherapeutic vaccine against Trypanosoma cruzi infection. Hum Vaccin Immunother.

[11] Bontempi IA, Vicco MH, Cabrera G, Villar SR, Gonzalez FB, Roggero EA, Ameloot P, Callewaert N, Perez AR, Marcipar IS (2015). Efficacy of a trans-sialidase-ISCOMATRIX subunit vaccine candidate to protect against experimental Chagas disease. Vaccine.

[12] Rodriguez-Morales O, Monteon-Padilla V, Carrillo-Sanchez SC, Rios-Castro M, Martinez-Cruz M, Carabarin-Lima A, Arce-Fonseca M (2015). Experimental Vaccines against Chagas Disease: A Journey through History. J Immunol Res.

[13] Lantos AB, Carlevaro G, Araoz B, Ruiz Diaz P, Camara Mde L, Buscaglia CA, Bossi M, Yu H, Chen X, Bertozzi CR, Mucci J, Campetella O (2016). Sialic Acid Glycobiology Unveils Trypanosoma cruzi Trypomastigote Membrane Physiology. PLoS Pathog.

[14] Dc-Rubin SS, Schenkman S (2012). Trypanosoma cruzi trans-sialidase as a multifunctional enzyme in Chagas’ disease. Cell Microbiol.

[15] Fontanella GH, De Vusser K, Laroy W, Daurelio L, Nocito AL, Revelli S, Contreras R (2008). Immunization with an engineered mutant trans-sialidase highly protects mice from experimental Trypanosoma cruzi infection: a vaccine candidate. Vaccine.

[16] Hoft DF, Eickhoff CS, Giddings OK, Vasconcelos JR, Rodrigues MM (2007). Trans-sialidase recombinant protein mixed with CpG motif-containing oligodeoxynucleotide induces protective mucosal and systemic trypanosoma cruzi immunity involving CD8+ CTL and B cell-mediated cross-priming. J Immunol.

[17] Arce-Fonseca M, Ballinas-Verdugo MA, Zenteno ER, Suarez-Flores D, Carrillo-Sanchez SC, Alejandre-Aguilar R, Rosales-Encina JL, Reyes PA, Rodriguez-Morales O (2013). Specific humoral and cellular immunity induced by Trypanosoma cruzi DNA immunization in a canine model. Vet Res.

[18] Costa F, Pereira-Chioccola VL, Ribeirao M, Schenkman S, Rodrigues MM (1999). Trans-sialidase delivered as a naked DNA vaccine elicits an immunological response similar to a Trypanosoma cruzi infection. Braz J Med Biol Res.

[19] Hoft DF, Eickhoff CS (2002). Type 1 immunity provides optimal protection against both mucosal and systemic Trypanosoma cruzi challenges. Infect Immun.

[20] Dumonteil E (2007). DNA Vaccines against Protozoan Parasites: Advances and Challenges. J Biomed Biotechnol.

[21] Dumonteil E, Escobedo-Ortegon J, Reyes-Rodriguez N, Arjona-Torres A, Ramirez-Sierra MJ (2004). Immunotherapy of Trypanosoma cruzi infection with DNA vaccines in mice. Infect Immun.

[22] Rodrigues MM, Ribeirao M, Pereira-Chioccola V, Renia L, Costa F (1999). Predominance of CD4 Th1 and CD8 Tc1 cells revealed by characterization of the cellular immune response generated by immunization with a DNA vaccine containing a Trypanosoma cruzi gene. Infect Immun.

[23] Ruiz Diaz P, Mucci J, Meira MA, Bogliotti Y, Musikant D, Leguizamon MS, Campetella O (2015). Trypanosoma cruzi trans-sialidase prevents elicitation of Th1 cell response via interleukin 10 and downregulates Th1 effector cells. Infect Immun.

[24] Ndure J, Flanagan KL (2014). Targeting regulatory T cells to improve vaccine immunogenicity in early life. Front Microbiol.

[25] Lacan G, Dang H, Middleton B, Horwitz MA, Tian J, Melega WP, Kaufman DL (2013). Bacillus Calmette-Guerin vaccine-mediated neuroprotection is associated with regulatory T-cell induction in the 1-methyl-4-phenyl-1,2,3,6-tetrahydropyridine mouse model of Parkinson's disease. J Neurosci Res.

[26] Macatangay BJ, Szajnik ME, Whiteside TL, Riddler SA, Rinaldo CR (2010). Regulatory T cell suppression of Gag-specific CD8 T cell polyfunctional response after therapeutic vaccination of HIV-1-infected patients on ART. PLoS One.

[27] Moore AC, Gallimore A, Draper SJ, Watkins KR, Gilbert SC, Hill AV (2005). Anti-CD25 antibody enhancement of vaccine-induced immunogenicity: increased durable cellular immunity with reduced immunodominance. J Immunol.

[28] Hori S, Sakaguchi S (2004). Foxp3: a critical regulator of the development and function of regulatory T cells. Microbes Infect.

[29] Youn JI, Nagaraj S, Collazo M, Gabrilovich DI (2008). Subsets of myeloid-derived suppressor cells in tumor-bearing mice. J Immunol.

[30] Arocena AR, Onofrio LI, Pellegrini AV, Carrera Silva AE, Paroli A, Cano RC, Aoki MP, Gea S (2014). Myeloid-derived suppressor cells are key players in the resolution of inflammation during a model of acute infection. Eur J Immunol.

[31] Goni O, Alcaide P, Fresno M (2002). Immunosuppression during acute Trypanosoma cruzi infection: involvement of Ly6G (Gr1(+))CD11b(+ )immature myeloid suppressor cells. Int Immunol.

[32] Mariano FS, Gutierrez FR, Pavanelli WR, Milanezi CM, Cavassani KA, Moreira AP, Ferreira BR, Cunha FQ, Cardoso CR, Silva JS (2008). The involvement of CD4+CD25+ T cells in the acute phase of Trypanosoma cruzi infection. Microbes Infect.

[33] Kotner J, Tarleton R (2007). Endogenous CD4(+) CD25(+) regulatory T cells have a limited role in the control of Trypanosoma cruzi infection in mice. Infect Immun.

[34] Sales PA, Golgher D, Oliveira RV, Vieira V, Arantes RM, Lannes-Vieira J, Gazzinelli RT (2008). The regulatory CD4+CD25+ T cells have a limited role on pathogenesis of infection with Trypanosoma cruzi. Microbes Infect.

[35] Gonzalez FB, Villar SR, Fernandez Bussy R, Martin GH, Perol L, Manarin R, Spinelli SV, Pilon C, Cohen JL, Bottasso OA, Piaggio E, Perez AR (2015). Immunoendocrine dysbalance during uncontrolled T. cruzi infection is associated with the acquisition of a Th-1-like phenotype by Foxp3(+) T cells. Brain Behav Immun.

[36] de Araujo FF, Correa-Oliveira R, Rocha MO, Chaves AT, Fiuza JA, Fares RC, Ferreira KS, Nunes MC, Keesen TS, Damasio MP, Teixeira-Carvalho A, Gomes JA (2012). Foxp3+CD25(high) CD4+ regulatory T cells from indeterminate patients with Chagas disease can suppress the effector cells and cytokines and reveal altered correlations with disease severity. Immunobiology.

[37] de Araujo FF, Vitelli-Avelar DM, Teixeira-Carvalho A, Antas PR, Assis Silva Gomes J, Sathler-Avelar R, Otavio Costa Rocha M, Eloi-Santos SM, Pinho RT, Correa-Oliveira R, Martins-Filho OA (2011). Regulatory T cells phenotype in different clinical forms of Chagas’ disease. PLoS Negl Trop Dis.

[38] Makino T, Skretas G, Georgiou G (2011). Strain engineering for improved expression of recombinant proteins in bacteria. Microb Cell Fact.

[39] Movahedi K, Guilliams M, Van den Bossche J, Van den Bergh R, Gysemans C, Beschin A, De Baetselier P, Van Ginderachter JA (2008). Identification of discrete tumor-induced myeloid-derived suppressor cell subpopulations with distinct T cell-suppressive activity. Blood.

[40] Mucci J, Risso MG, Leguizamon MS, Frasch AC, Campetella O (2006). The trans-sialidase from Trypanosoma cruzi triggers apoptosis by target cell sialylation. Cell Microbiol.

[41] Pitcovsky TA, Buscaglia CA, Mucci J, Campetella O (2002). A functional network of intramolecular cross-reacting epitopes delays the elicitation of neutralizing antibodies to Trypanosoma cruzi trans-sialidase. J Infect Dis.

[42] Chuenkova M, Pereira M, Taylor G (1999). trans-sialidase of Trypanosoma cruzi: location of galactose-binding site(s). Biochem Biophys Res Commun.

[43] Claser C, Espindola NM, Sasso G, Vaz AJ, Boscardin SB, Rodrigues MM (2007). Immunologically relevant strain polymorphism in the Amastigote Surface Protein 2 of Trypanosoma cruzi. Microbes Infect.

[44] Heithoff DM, Enioutina EY, Bareyan D, Daynes RA, Mahan MJ (2008). Conditions that diminish myeloid-derived suppressor cell activities stimulate cross-protective immunity. Infect Immun.

[45] Martino A, Badell E, Abadie V, Balloy V, Chignard M, Mistou MY, Combadiere B, Combadiere C, Winter N (2010). Mycobacterium bovis bacillus Calmette-Guerin vaccination mobilizes innate myeloid-derived suppressor cells restraining in vivo T cell priming via IL-1R-dependent nitric oxide production. J Immunol.

[46] Centuori SM, Trad M, LaCasse CJ, Alizadeh D, Larmonier CB, Hanke NT, Kartchner J, Janikashvili N, Bonnotte B, Larmonier N, Katsanis E (2012). Myeloid-derived suppressor cells from tumor-bearing mice impair TGF-beta-induced differentiation of CD4+CD25+FoxP3+ Tregs from CD4+CD25-FoxP3- T cells. J Leukoc Biol.

[47] Luan Y, Mosheir E, Menon MC, Wilson D, Woytovich C, Ochando J, Murphy B (2013). Monocytic myeloid-derived suppressor cells accumulate in renal transplant patients and mediate CD4(+) Foxp3(+) Treg expansion. Am J Transplant.

[48] Nardy AF, Freire-de-Lima CG, Perez AR, Morrot A (2016). Role of Trypanosoma cruzi Trans-sialidase on the Escape from Host Immune Surveillance. Front Microbiol.

[49] Eickhoff CS, Zhang X, Vasconcelos JR, Motz RG, Sullivan NL, O’Shea K, Pozzi N, Gohara DW, Blase JR, Di Cera E, Hoft DF (2016). Costimulatory Effects of an Immunodominant Parasite Antigen Paradoxically Prevent Induction of Optimal CD8 T Cell Protective Immunity. PLoS Pathog.

[50] Sambrook J, Fritsch E. F, Maniatis T (1989). Molecular Cloning: A Laboratory Manual.

[51] Bainor A, Chang L, McQuade TJ, Webb B, Gestwicki JE (2011). Bicinchoninic acid (BCA) assay in low volume. Anal Biochem.

