# Supplementary material for: Trans-sialidase-based vaccine candidate protects against Trypanosoma cruzi infection, not only inducing an effector immune response but also affecting cells with regulatory/suppressor phenotype
Source: Oncotarget. 2017 May 25;8(35):58003–20. doi: 10.18632/oncotarget.18217 (PMC5601629; doi:10.18632/oncotarget.18217)
Supplement: Supplementary file 1 [file oncotarget-08-58003-s001.pdf]

Trans-sialidase-based vaccine candidate protects against *Trypanosoma cruzi* infection, not only inducing an effector immune response but also affecting cells with regulatory/ suppressor phenotype

Supplementary Material

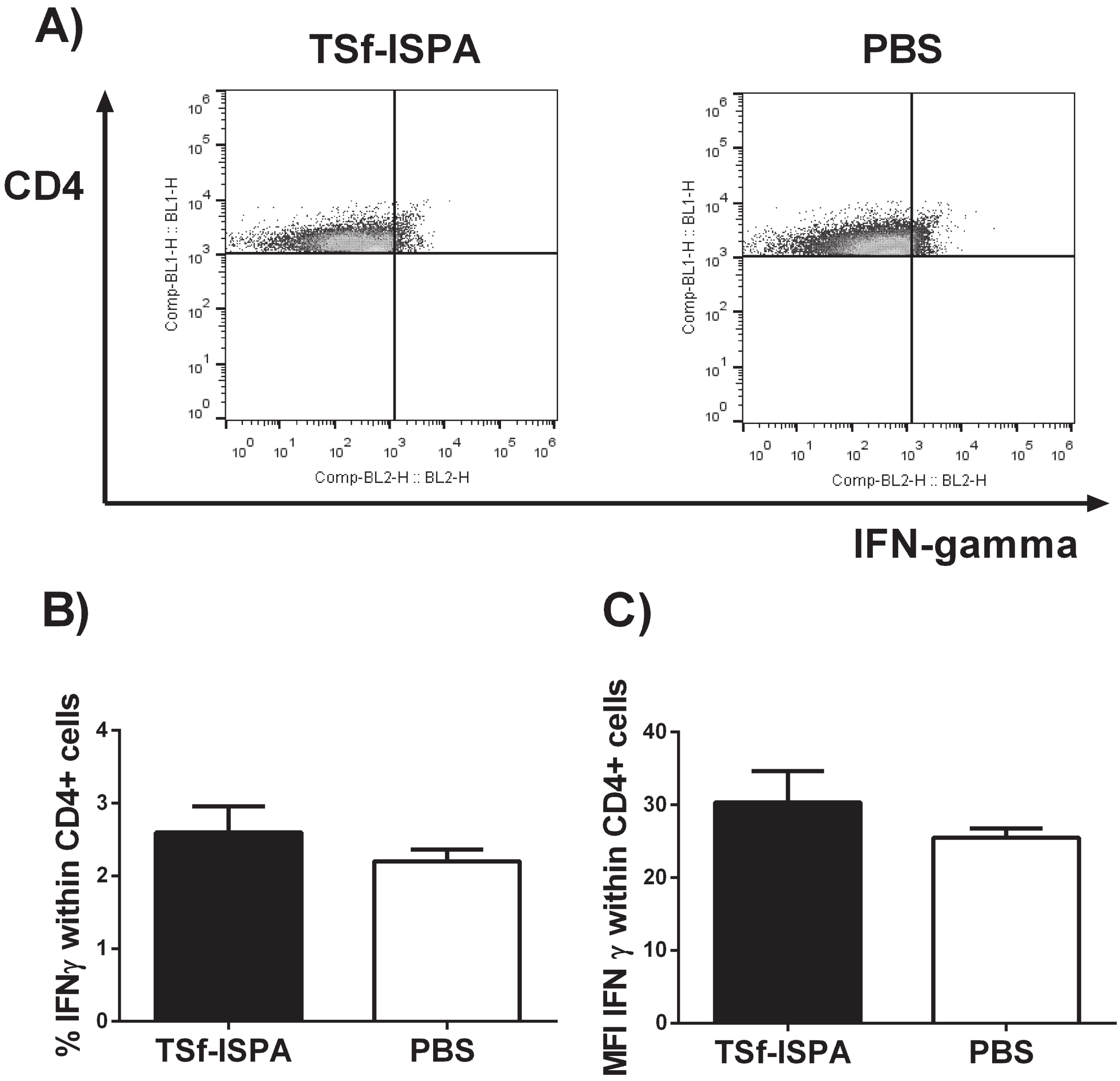

Supplementary Figure 1: Production of IFN-γ within CD4+ cultured cells. A) Representative dot plots of CD4+ splenocytes from TSf-ISPA immunized and PBS-inoculated mice cultured in the presence of *T. cruzi* homogenate. B) Splenocytes from TSf-ISPA immunized mice showed a slight but not significant increase in the production of IFN-γ within CD4+ cultured cells, C) Splenocytes from TSf-ISPA immunized mice showed a slight but not significant increase in the MFI of IFN-γ within CD4+ cultured. Data are expressed as means + standard deviations. Results shown are representative of 2-3 independent experiments (n = 4-10 mice per group), $^*p < 0.05$, Mann-whitney test.

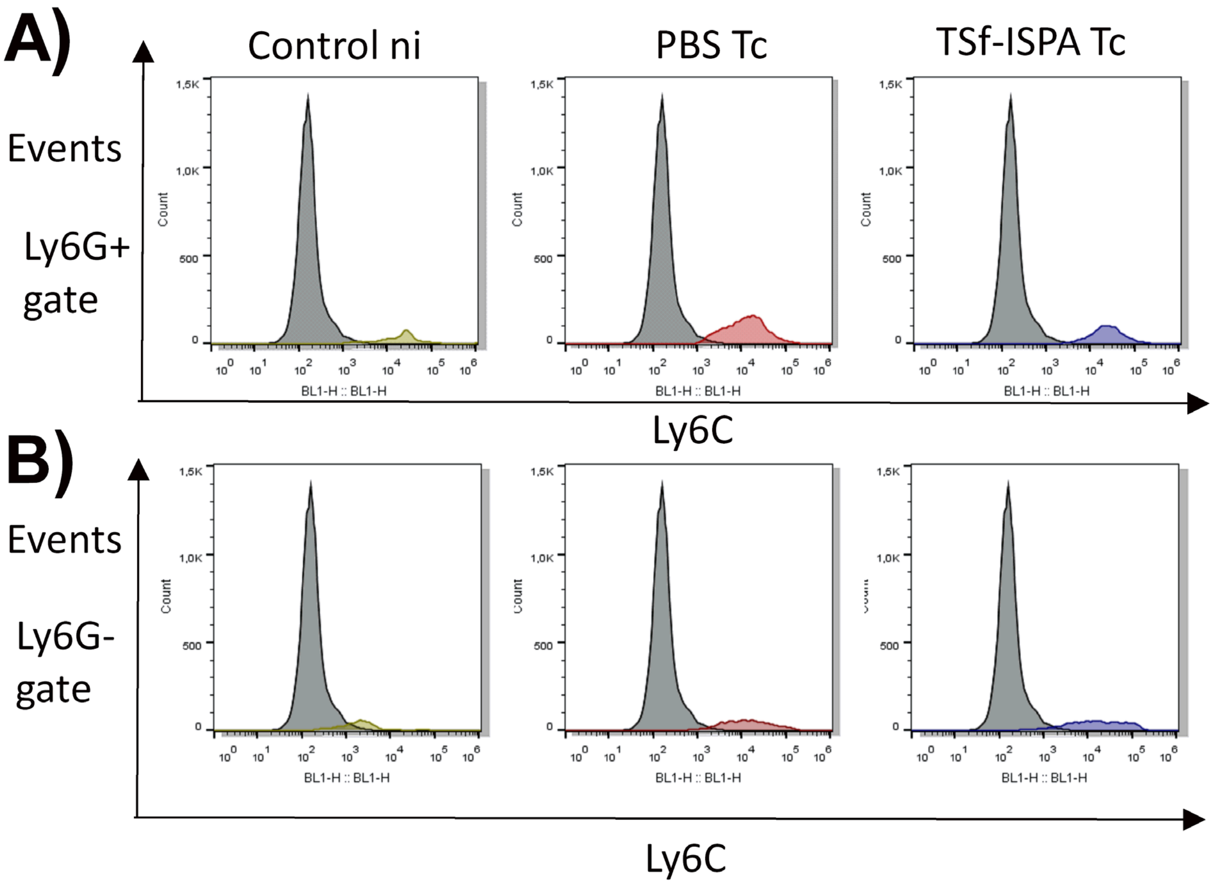

Supplementary Figure 2: Analysis of Ly6C+ expression within Ly6G+ cells in the spleen at day 21 post T. cruzi infection. Splenocyte suspensions were prepared and expression of Ly6C-FITC, Ly6G-PE and CD11b-PerCP-Cy5.5 expression was analyzed by flow cytometry in control non-infected mice (control), PBS-inoculated and infected mice (PBS Tc) and TSf-ISPA immunized and infected mice (TSf-ISPA). A) and B Representative dot plots showing that both CD11b+ Ly6G+ and CD11b+Ly6G- gated cells express the Ly6C marker
